# Exploring auditory morphodynamics: Audiovisual associations in sound-based music

**DOI:** 10.1177/20416695251338718

**Published:** 2025-06-30

**Authors:** Riccardo Wanke, Alessandro Ansani, Nicola Di Stefano, Charles Spence

**Affiliations:** University of Coimbra, Portugal; University of Jyväskylä, Finland; National Research Council of Italy (CNR), Italy; University of Oxford, UK

**Keywords:** audiovisual associations, crossmodality, crossmodal correspondences, sound-based music, morphodynamic theory

## Abstract

This article explores audiovisual associations within the context of contemporary and experimental music practices, particularly focusing on sound-based music. While extensive studies exist on crossmodality in relation to traditional music genres (such as classical instrumental music), the perceptual potential of sound-based music remains an underexplored field of psychological research. In an online procedure, 152 participants were exposed to six musical excerpts from spectralism and electronic-glitch music and were asked to rate the extent to which each audio matched with six ad hoc generated black and white abstract images. Statistical analysis revealed that ratings were highly consistent across participants, indicating that they may rely on a shared set of implicit perceptual criteria rooted in Gestalt and morphodynamic features common to both auditory and visual stimuli. In particular, smoothness, continuity, numericity, symmetry, and spectrotemporal dimensions emerged as the primary factors influencing the association ratings. We discuss the implication of these findings both for crossmodal research and musicology, and suggest some directions for future research in audiovisual associations using sound-based music.

## How to cite this article

Wanke, R., Ansani, A., Di Stefano, N., & Spence, C., (2025). Exploring auditory morphodynamics: Audiovisual associations in sound-based music. *i-Perception, 16*(4), 1–20. https://doi.org/10.1177/20416695251338718

Crossmodal correspondences (or associations), defined as the consensual associations between stimuli or sensory features across different senses observed in normal perceivers (Spence, 2011), have been increasingly studied in perception science over the past decade or two. Observed between all of the spatial (vision, touch, hearing) and chemical (smell, taste) senses, crossmodal associations reflect one of the putative mechanisms by which our sensory systems can integrate diverse stimuli into coherent constructs. Such research thus connects with foundational questions in multisensory integration and its implications for emotion and learning (Spence, 2011). Scholars have elaborated several explanations to account for crossmodal associations (see Motoki et al., 2023), ranging from statistical learning (Spence, 2011) to emotional mediation hypothesis (Spence, 2020) and amodal representations theory (Marks, 1978, though see Spence & Di Stefano, 2024, for a critical review of the notion of amodal sensory dimensions).

Pioneering approaches to audiovisual associations were prompted by early Gestalt psychologists’ exploration of the association between certain sounds and geometrical shapes. Those studies demonstrated that certain visual forms (e.g., jagged or rounded shapes) were consistently associated with specific words or phonemes (i.e., the buba-kiki effect, Bremner et al., 2013; Eitan, 2013; Köhler, 1929; Leech-Wilkinson & Prior, 2017; Ramachandran & Hubbard, 2001; see also Ćwiek et al., 2024). More recently, experimental approaches have revealed that, while participants’ associations can be influenced by cultural cues (Küssner et al., 2014; Löbbers & Fazekas, 2021), the intrinsic properties of the sound samples play a significant role in audiovisual matching tasks, especially when certain musical styles or genres are adopted (e.g., Di Stefano et al., 2024, 2025). For example, the impact of sonic characteristics is evident in audiovisual art forms and multimedia contexts, where music and images are often deliberately combined to evoke specific emotions or narrative coherence (Ansani et al., 2020; Cohen, 2013; Herget, 2019; Steffens, 2020), leveraging our innate tendency for multisensory integration.

Within the broad panorama of studies on crossmodal associations between music and shapes, the relationships between music styles and types of shapes, and the related question of which kinds of sound patterns are preferentially associated to a certain kind of images, remain largely underexplored. Moreover, in the field of music psychology, only a relatively small number of studies have considered contemporary art music (Deil et al., 2022; Mencke et al., 2019; Ockelford & Sergeant, 2012). This is somewhat surprising, given that experimental music practices may have strong potential to elicit specific kinds of visual imagery (Markert & Küssner, 2021), particularly in relation to the sonic features of these music genres. Notably, the listening experience of contemporary and experimental music performances typically includes immersive environments and three-dimensional diffusion, virtual realities, wavefield synthesis and multi-modal experiences. Moreover, the sonic material itself of these styles of music extends to broad frequency ranges, noises, electronic and real-world sounds, using extreme timbral contrasts, or aural stimuli at the threshold of our perception and being characterised by non-teleological constructions, non-linear temporalities and metrics (Solomos, 2019; Wanke, 2021). These specific traits likely have an influence on the impact of such music which, it may be expected, operate differently insofar as it confronts the listener with a wider range of sounds and evolutions compared to the more restricted set of conventional musical sounds (Grisey, 1987; Imberty, 2005).

This study aims to fill this gap in the literature, by exploring a particular group of music genres typically considered as sound-based music (Landy, 2007; Wanke, 2023a), namely electroacoustic music, mixed-source works, post-spectralism, glitch-electronica, and various areas of sound art. Works within each of these genres show a range of characteristics such that, for example, not all electroacoustic music fits the “sound-based” label.^1^ Works that fit this label typically do not focus on traditional compositional organisations (e.g., melodies) or functional interplays (e.g., counterpoint), rather they are grounded on the intrinsic properties of sound and the creation of sonic textures and masses in motion. This cross-genre area of music has recently become more distinct due to its specific sonic features and thus calls for new theoretical and aesthetic perspectives (Solomos, 2019; Wanke, 2021).

For our purposes, the key feature of sound-based music is that it is essentially perceived as a sequence of sound patterns which prompt a phenomenological and sensory engagement rather than a cognitively mediated response, such as episodic or autobiographical memories (Schrimshaw, 2017; Wanke, 2021). Rather than developing culturally articulated musical figures, sound-based music appears in fact to be rooted in processing mechanisms of early stages of perception, such as primal segregation (Bregman, 1990) and auditory Gestalten formation (Koelsch & Siebel, 2005, p. 579). These essentials function as temporal spans, or “temporal Gestalt-units” (Tenney & Polansky, 1980, p. 205), that embody essential dichotomies (e.g., identity/difference, movement/stasis), spatial organisations (e.g., ascending/descending, figure/background), and scalar and kinaesthetic dimensions (e.g., tension, linearity, amplitude, and projection; Van de Cruys & Wagemans, 2011). Sound-based music exposes listeners to a series of stimuli which bring to mind a set of geometries—lines, planes, layers, 3D shapes—in motion (Wanke, 2023a). Some works (Godøy, 1997; Reybrouck, 2013) have already put forward the idea of a shape-paradigm which may have an important connection with our bodily responses, and others elaborated on the possibility of seeing the perceptual experience of sound-based music in terms of a morphodynamic link between the sound patterns and our phenomenological responses (Wanke, 2023b). Findings have revealed a general preference among listeners for the linear spectrotemporal visual representation of their listening experience (Wanke, 2023b), coherently with findings showing that sound patterns for which no intuitive causal actions can be identified (as is the case for the sound-based music considered here) can trigger movements that reflect spectrotemporal contours (Caramiaux et al., 2014).

Taking into account all of the above aspects, we designed a survey to explore how listeners match visual stimuli with sound-based music and what are the perceptual factors participants rely on to assess the matchings.

## Method

### Participants

One hundred fifty-two participants took part in the study (53 male, 95 female, four did not specify, age: *M* = 27.8 years, *SD* = 7.8). Only 21% (14 male, 18 female) reported a prior professional (or semi-professional) experience with music through being either students of music or musicology, musicians, or scholars, and 79% of the participants (39 male, 77 female, four did not specify) declared themselves to be musically “untrained.” The degree of familiarity with the musical excerpts has also been assessed: 36% (19 male, 34 female, one did not specify) reported a good or high familiarity with the music, 42% (20 male, 41 female, three did not specify) were familiar with just some of the audio samples, and 22% of the participants (14 male, 20 female) declared that they had very little (did not have any) familiarity.

### Procedure

The survey was carried out through a web-based platform (Prolific.com). After some general cultural/demographic questions (age, link to music, musical preferences, and familiarity with the audio samples^2^), participants completed the perceptual task, during which they listened to six audio samples while six images were displayed on the screen. For each audio sample, participants were asked to evaluate the degree of correspondence (i.e., fit score)—using a Visual Analogue Scale from 0 (“*no association*”) to 100 (“*total association*”)—with each of the six images. The participants had the possibility to add free comments in a box. The procedure ended with questions about the listener's personal opinion about the audio samples, the drawings and the questionnaire itself in order to obtain insights concerning the level of interest of the participant for the present investigation and possible suggestions for improvement. The procedure lasted 758 s (12–13 mins) on average ([95% CI: 693, 823]; *SD* = 405). Ethical approval was obtained from the CEIIIIUC of the University of Coimbra (n. 46_ID1962).

### Stimuli Design

#### Images

Six images consisting of abstract shapes were prepared by the first author to visually match with the morphologies, discontinuities, and textures of audio samples (see Table 1). The criteria that inspired the generation of the images included continuity, smoothness, numericity, and verticality in the frequency domain (Cox, 2016; Küssner, 2017). Each image has been deliberately created to better fit with one single audio sample.

**Table 1.** Details of the audio stimuli, their musical features, Gestalt principles and images that were generated to mirror the auditory stimuli.

#	Audio stimuli	Musical features	Gestalt principles	Images	
1	Ligeti—*Atmosphères* [03’02’’–03’37’’, bars 31–37, duration 35 s]	Orchestral pitch rising	Continuity, progressive change, common fate	A	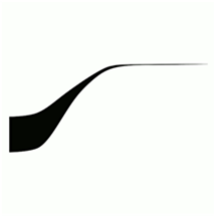
2	Demdike Stare—*New Fakes* [01’12’’–01’48’’, duration 36 s]	Distorted bass electronic drone (fade in/out structure)	Continuity, cyclic structure, progressive change	B	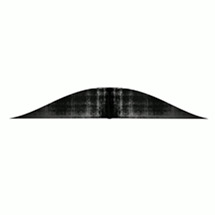
3	Ligeti—*Ramifications* (Version für 12 Solostreicher) [01’10’’–01’46’’, duration 36 s]	Continuous flow of repeated up-down tone movements	Continuity, stability, regularity	C	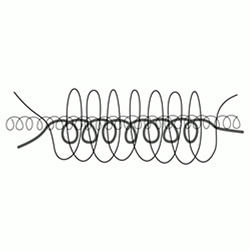
4	Haas—*String Quartet n°2* [15’30’’–16’06’’, duration 36 s]	Continuous flow of multiple tones in parallel	Continuity, common fate	D	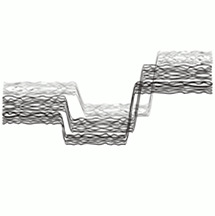
5	Emptyset—*Descent* [00’00’’–00’36’’, duration 36 s]	Pulses of bass distorted electronic sounds	Fragmentation, repetition	E	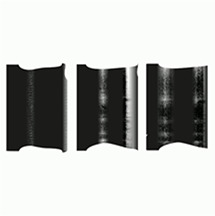
6	Ligeti—*Atmosphères* [03’36’’–04’03’’, bars 37-42, duration 27 s]	Continuous high pitched orchestral sound that abruptly jumps to lower pitch drone (mainly of strings).	Continuity, change/interruption, common fate	F	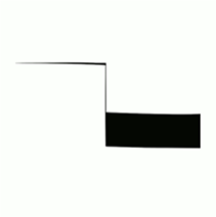

#### Musical Stimuli

Six audio excerpts (Table 1) were selected based upon previous studies (Wanke, 2018, 2023a). The audio stimuli were fed as .wav files (44.1 kHz, 16-bit, stereo). The compositions belong to specific styles of experimental music, namely spectralism (Ligeti, Haas), and electronic-glitch music (Demdike Stare, Emptyset). The compositions were:

1. Ligeti—*Atmosphères* (Ligeti, 1988);
2. Demdike Stare—*New Fakes* (Whittaker & Canty, 2018);
3. Ligeti—*Ramifications* (Version für 12 Solostreicher) (Ligeti, 1988);
4. Haas—*String Quartet n°2* (Haas, 1998);
5. Emptyset—*Descent* (Ginzburg & Purgas, 2017);
6. Ligeti—*Atmosphères* (Ligeti, 1988).

The selection was made in order to present listeners with events consisting of sound patterns whose characteristics align well with the criteria that inspired image creation, for example, the Gestalt principles (Wanke, 2023a, 2023b) of continuity/discontinuity, smoothness/sharpness, common fate, and repetition (numericity, the identification of a certain number of entities). Table 1 presents the hypothesised relationships between the principles, the musical excerpt and the visual stimuli.

### Statistical Analysis

The statistical analyses were carried out in the R environment (RStudio 2024.09.0, 375 “Cranberry Hibiscus” for macOS) via several packages: *lme4* (Bates et al., 2015), *glmmTMB* (Brooks et al., 2017), *DHARMa* (Hartig, 2024), *emmeans* (Lenth, 2017), *factoextra* (Kassambara & Mundt, 2016), and *easybgm* (Huth et al., 2023, 2024). The statistical analysis consists of two parts: in the first part (i.e., modelling), the fit scores assigned by the participants to the images were modelled in a Generalised Linear Mixed Model (GLMM) framework to inspect the association patterns and strength. In the second part, the relationships among the image scores were investigated using a Spearman Correlation Analysis and a Bayesian Network Analysis. A Principal Component Analysis of the fit scores can be found in the Supplemental Materials.

#### Modelling

Consistent with similar studies (Di Stefano et al., 2024, 2025), the fit scores were modelled via a GLMM as a function of the musical excerpts, visual images, and their interaction. To correct for the repeated-measure nature of the data, participants were modelled as random intercepts. Thus, the following formula was used:

fitscore=image×musicalexcerpt+(1|ID)

Given the high proportion of zeros in the fit scores, two model versions were constructed. The first used a Gaussian distribution (without any link function), which assumes a continuous response variable. The second model version used a zero-inflated Tweedie distribution (with a logarithmic link function), that is specifically designed to handle data with a combination of zeros and positive continuous values (Dunn & Smyth, 2018), better reflecting the nature of our dataset. Subsequently, the model fit of the two versions was compared in terms of Akaike and Bayesian Information Criteria (i.e., AIC and BIC), Conditional R^2^, and Mean Absolute Error (MAE). To ensure comparability, all models were estimated using Maximum Likelihood, which allows for valid comparisons of model fit indices such as AIC and BIC. A verification of the assumptions of the best-performing model was carried out (see Supplemental Figures S3, S4, S5). Additionally, as we were interested in checking whether the familiarity and the professional connection to music had some influence on the associations, we also fitted two models by adding those variables and a three-way interaction with the main interaction (i.e., Image × Musical Excerpt × Familiarity and Image × Musical Excerpt × Link to Music). We subsequently compared their fit indices to see whether such added variables contributed significantly. In the best-fitting model, the fit score of each image within each musical excerpt was contrasted against the grand mean of all other images within the same musical excerpt (i.e., deviation contrast), and the significance level was adjusted via Bonferroni correction for the number of tests (i.e., 36). The difference against the grand mean is reported in the model’s results as *Δ_GM_* next to its *p*-value.

#### Effect Size Calculation and Sensitivity Analysis

We calculated the effect sizes for the within-subject deviation contrasts derived from the Gaussian model using Cohen’s *d*. Specifically, we estimated *d* by dividing the contrast estimates from the model by the residual standard deviation (σ), which accounts for the variance in the data while considering the model’s fixed and random effects structure. To perform a sensitivity analysis, we approximated the required effect size for achieving 80% power using a paired *t*-test. This approximation assumes that the within-subject design of the contrasts is comparable to paired differences, enabling us to compute the Minimum Detectable Effect Size (MDES) given our sample size (*N* = 152) and an α level of .05. The sensitivity analysis results indicate that our sample was adequate; namely, it was sufficient to detect deviation contrast effect sizes as low as *d* = ±0.23, that is, a small effect.

#### Spearman Correlation Analysis and Bayesian Network Analysis

To conduct a thorough examination of the relationships between the image scores, we used both Spearman correlation matrix analysis and Bayesian Network Analysis. We included both these methods because they provide complementary perspectives. The correlation matrix offers a simple and direct measure of pairwise associations between the fit scores, showing how strongly two variables are related without considering the influence of others. This is useful for identifying general patterns of shared variance. However, mere correlation matrices do not distinguish between direct connections and those mediated by other associations, potentially leading to overestimation of certain relationships. In contrast, Network Analysis focuses on conditional dependencies, highlighting direct and unique relationships between image scores while accounting for the influence of all other associations in the network.

Both analyses were carried out on the fit scores assigned to the images for all musical excerpts. To account for the repeated-measures nature of the data and ensure an equal contribution from all participants, the fit scores were z-transformed within each participant before the analysis. The network structure consists of unweighted, undirected edges, where an edge’s presence denotes conditional dependence between the scores of two images, while its absence indicates conditional independence (Huth et al., 2024).^3^ The estimate of the edge’s weight corresponds to a partial correlation coefficient that ranges in the interval [−1, 1]; namely, the correlation between the scores of two images controlling for all other associations in the network. In other words, by controlling for all possible associations in the network, we acknowledge that each partial correlation coefficient does not represent the similarity between the visual stimuli *per se*. Instead, it reflects the similarity in the association profiles between two images based on how the participants linked the images to the particular set of musical stimuli.

Importantly, in the Bayesian framework, the strength of evidence for both the presence and absence of a particular edge can be assessed. In this case, a network was fit via 150 million MCMC sampling iterations, and the prior inclusion probability of each edge was set to 50%, to reflect a neutral stance. Subsequently, an inclusion/exclusion Bayes Factor (i.e., *BF*) was computed for each edge. Consistent with commonly used thresholds, a *BF* > 10 (i.e., strong evidence) was interpreted as evidence for an edge's presence, whereas a *BF* < 0.10 indicated evidence for its absence (Wagenmakers et al., 2011). As in our design each musical excerpt corresponded to one image, we expect that, after controlling for all the other associations in the network, all edges (i.e., partial correlations) will be negative or non-existent.

## Results

### Modelling

#### Model Comparison

In terms of information criteria and *R*^2^, the Tweedie model performed considerably better. Indeed, the drops in the AIC and BIC values were respectively ΔAIC = 6909 and ΔBIC = 6902, exceeding the commonly used thresholds for practical equivalence (Burnham & Anderson, 2010, p. 71). Furthermore, the Conditional *R*^2^ of the Tweedie model increased by Δ*R*^2^ = 0.076, thus explaining 29.80% more variance than the Gaussian model. When looking at the MAE, the values were indistinguishable (see Table 2). At this point, we fitted a third Tweedie model by adding the “familiarity” variable and a fourth model with the “professional connection to music” variable, with the three-way interactions described above. Two Likelihood Ratio Tests confirmed that none of these models improved the fit significantly (*p*_familiarity_ = .816; *p*_connectiontomusic_ = .845). Furthermore, Bayes Factors computed via BIC approximation (Wagenmakers, 2007) revealed that the evidence for the simpler model was extreme in both cases (*BF*_simple−familiarity_ = 1.38 × 10^61^; *BF*_simple−connectiontomusic_ = 2.10 × 10^61^). These findings suggest that neither the familiarity with, nor the professional connection to music had an impact on the associations. All this considered, we describe the results of the Tweedie model right after the evaluations of their assumptions.

**Table 2.** Model fit indices.

Model	Conditional R^2^	AIC	BIC	MAE
Gaussian	0.255	52,158	52,409	22.06
Tweedie	0.331	45,249	45,507	22.62
Tweedie + Fam	0.336	45,293	45,789	22.51
Tweedie + CtM	0.336	45,294	45,790	22.57

MAE = Mean Absolute Error; AIC = Akaike Information Criteria; BIC = Bayesian Information Criteria.

#### Model Assumptions

First, the randomised quantile residuals were computed and examined. The resulting plots (see Supplemental Materials) showed no systematic patterns or substantial deviations from the expected distribution, indicating that the model adequately captures the data. As a second step, we conducted an overdispersion test using the mean Pearson chi-square statistic. The result (dispersion = 0.96, df = 5433, *p* = .984) indicates no overdispersion, suggesting that the model appropriately accounts for variance in the data. Lastly, we tested for potential zero-inflation using the DHARMa zero-inflation test^4^ (Hartig, 2024). The observed-to-expected zero ratio (1.06, *p* = .088) suggests no strong evidence of excess zeros, indicating that the model adequately accounts for zero values in the data.

#### Model Results

Musical excerpt 1, from Ligeti’s *Atmosphères*, was mostly associated with A image (*Δ_GM_* = 24.33, *p* < .001, *d* = 0.88), as expected. The association with the D image was also noteworthy (*Δ_GM_* = 12.63, *p* < .001, *d* = 0.45), while matchings with images E (*Δ_GM_* = −10.81, *p* < .001, *d* = −0.39) and F (*Δ_GM_* = −14.60, *p* < .001, *d* = −0.53) were significantly below the grand mean.

Musical excerpt 2 (Demdike Stare,*New Fakes*) was significantly associated with Image B (*Δ_GM_* = 13.06, *p* < .001, *d* = 0.46), as expected; however, the strongest association was with Image C (*Δ_GM_* = 20.11, *p* < .001, *d* = 0.72). Images F (*Δ_GM_* = −13.19, *p* < .001, *d* = −0.48) and A (*Δ_GM_* = −7.34, *p* = .034, *d* = −0.27) presented significantly lower associations, whereas the association with Image D was slightly above the grand mean (*Δ_GM_* = 8.04, *p* = .009, *d* = 0.28).

Musical excerpt 3, taken from Ligeti’s *Ramifications*, was consistently associated with the hypothesised Image, C (*Δ_GM_* = 20.28, *p* < .001, *d* = 0.73) which captures a clear set of characteristics: continuity, smoothness, regularity, cyclicity, recurrence, and layering. Images F (*Δ_GM_* = −14.60, *p* < .001, *d* = −0.53) and D (*Δ_GM_* = −7.79, *p* = .014, *d* = −0.29) were negatively associated with it, whereas all other images didn’t diverge from the grand mean significantly.

Musical excerpt 4 (Haas, *String Quartet n°2*) was associated with the corresponding image, D (*Δ_GM_* = 8.32, *p* = .005, *d* = 0.29) and negatively associated with Image C (*Δ_GM_* = −8.28, *p* = .006, *d* = −0.12), all other associations being not significant.

Musical excerpt 5’s association (Emptyset, *Descent*) with its intended Image, E, was the strongest one (*Δ_GM_* = 28.20, *p* < .001, *d* = 1.02). The same excerpt was also mildly related to Image D (*Δ_GM_* = 7.33, *p* = .032, *d* = 0.26). Negative associations were found with images F (*Δ_GM_* = −12.26, *p* < .001, *d* = −0.45), B (*Δ_GM_* = −10.51, *p* < .001, *d* = −0.38), and A (*Δ_GM_* = −8.45, *p* = .004, *d* = −0.31).

**Figure 1.**
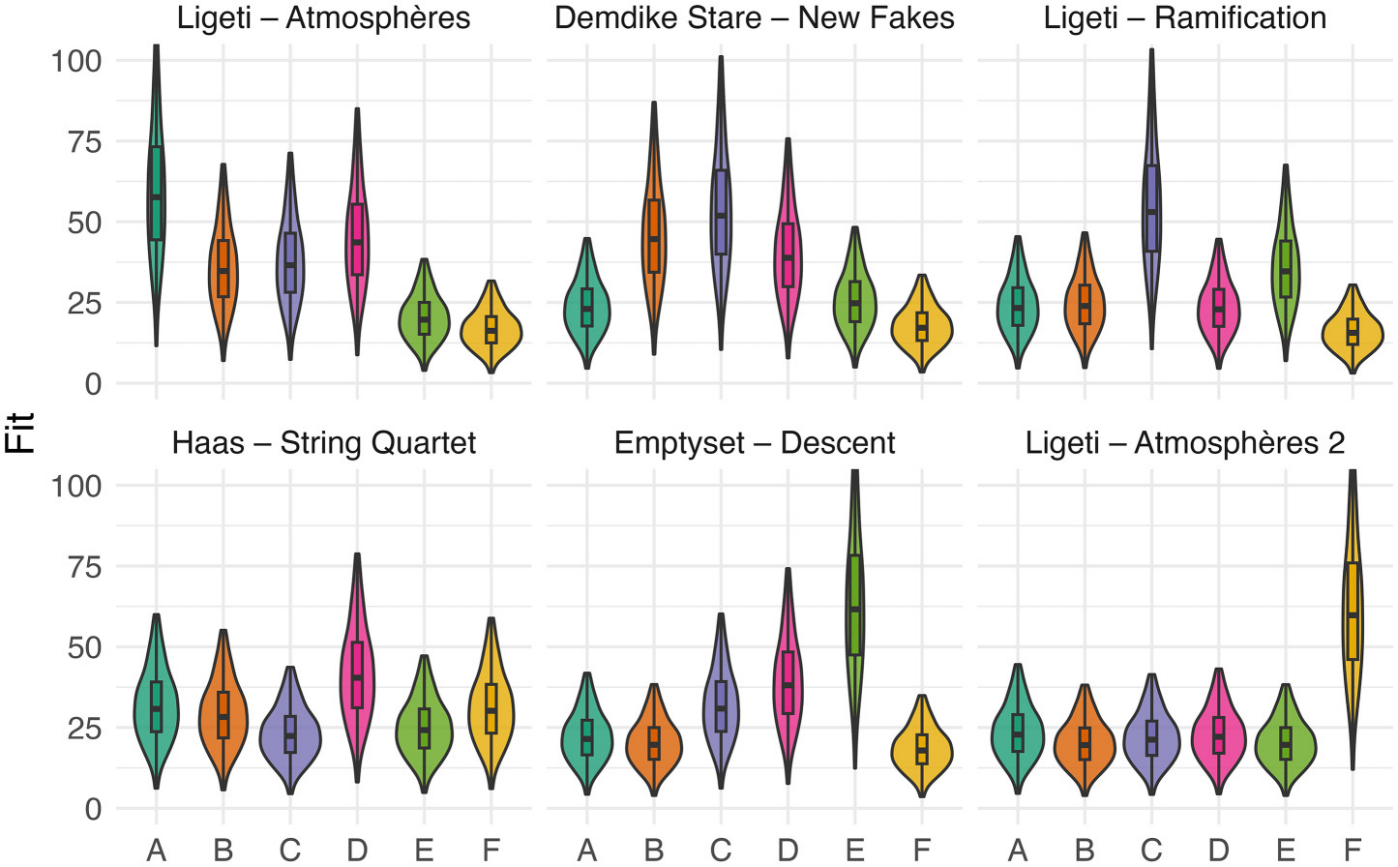
Violin plot of fit score across audio stimuli and images (letters). Violin plots visualise the data distribution by combining a kernel density plot (i.e., violin) with a boxplot. The shape of the violin represents the data’s distribution, while the boxplot within the violin shows the median and interquartile range. The values predicted by the Tweedie model are represented.

The pattern of excerpt 6, taken from Ligeti’s *Atmosphères*, was the clearest one. It was associated with its intended Image, F (*Δ_GM_* = 26.28, *p* < .001, *d* = 0.95) and negatively associated with all other images in a significant way (see Figure 1).

### Spearman Correlation Analysis

**Figure 2.**
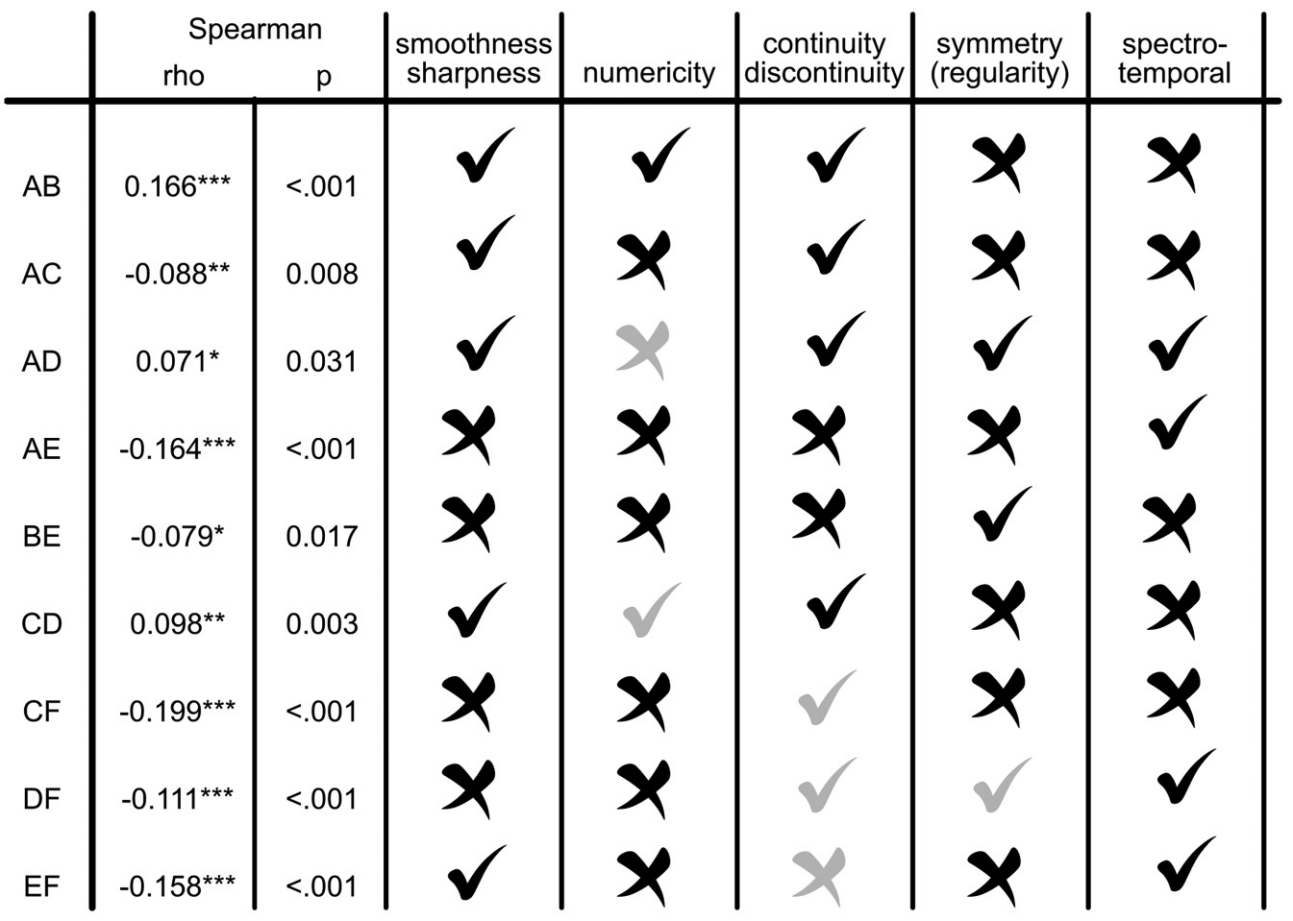
Correlation table and morphodynamic criteria (* *p* < .05, ** *p* < .01, *** *p* < .001). Grey marks indicate less relevant correlation. Image D, even if it could be considered a continuous single entity, it is visually divided in three steps. Therefore, in terms of numericity images A–D do not match, and C–D match. Image F is formally continuous but its sharpness weakens its continuous character: in the case of CF, DF, and EF this matching should be considered less relevant.

Examining Spearman’s coefficients, the most prominent correlations are the negative ones, as expected. Data from Principal Component Analyses (see Supplemental Materials) interestingly harmonise with the pairing of images based on the five morphological criteria listed above: smoothness/sharpness, continuity/discontinuity, numericity, spectrotemporal resemblance, symmetry/asymmetry (Figure 2). Then, smoothness/sharpness and numericity criteria appear the most relevant. A high negative Spearman’s coefficient is observed when these two criteria are not shared between the images: pairs such AE,^5^ CF, and DF are clearly opposite in PCA Biplot representation (see Supplemental Materials). Conversely, when both images exhibit smoothness but differ in numericity, other criteria—such as continuity, symmetry, and spectrotemporal profile—appear to play a more prominent role in explaining the correlation and PCA data.

**Figure 3.**
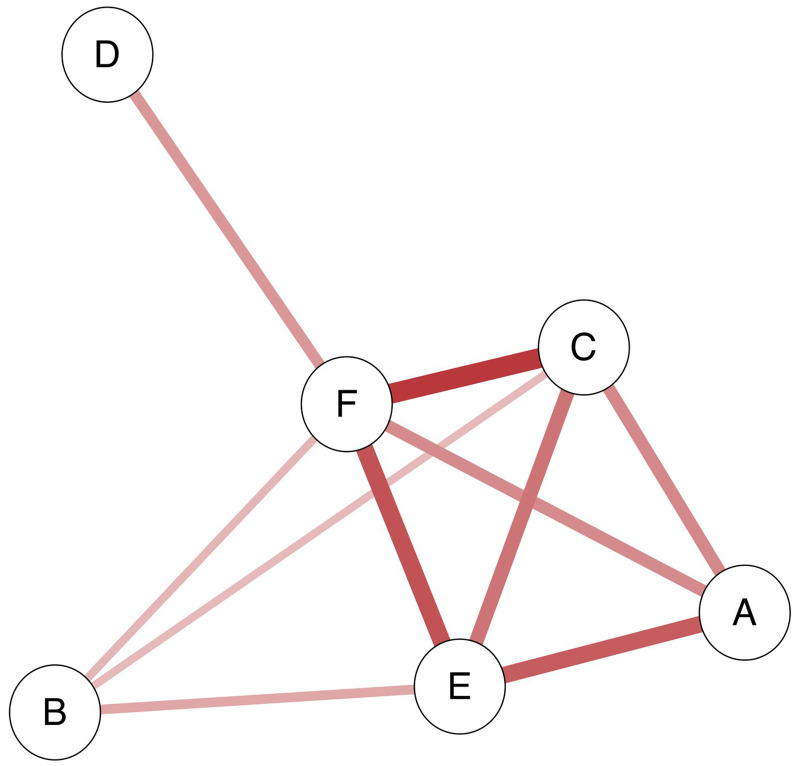
Bayesian network structure*.* The association plot shows all edges with an inclusion *BF* larger than 10. Edge thickness and saturation represent the strength of the association; the thicker the edge, the stronger the association. The dashed line represents the edge with substantial, but not strong evidence (i.e., *BF* = 9). The red colour indicates negative relations. Letters (A–F) refer to the images (see Table 1).

### Bayesian Network Analysis

Out of the 15 possible edges, we found sufficient evidence for inclusion (i.e., *BF* > 10) for nine of them. We also found adequate evidence for exclusion (i.e., *BF* < 0.10) for four edges. The two remaining edges had inconclusive results (i.e., *BF* = 0.19 and 9.00; see Table 3). A visual representation of the network is provided in Figure 3. The first notable observation is that, as expected, consistent with the idea that each image is paired with a specific musical excerpt, all correlation coefficients are either negative or non-existent. Image F shows a high degree of uniqueness as its score is negatively correlated with those of all other images. Similarly, Image E is negatively correlated with all other images except for D (*ρ* = −0.011, *BF* = 0.190). Image D scores showed evidence of no correlations with images A, B, and C, apart from the already described negative correlation with Image F. Image C’s negative relationships with images A, E, and F were confirmed, whereas its relationship with Image B meets the criterion for substantial evidence in favour of its presence (*ρ* = −0.105, *BF* = 9). Evidence of no relationship between images B and D was also found (*ρ* = −0.004, *BF* = 0.087). The only edges with inclusion evidence for Image B are those connecting it with F (*ρ* = −0.110, *BF* = 10.110) and E (*ρ* = −0.133, *BF* = 32.333). Lastly, Image A showed existent negative correlations with C (*ρ* = −0.179, *BF* > 100), E (*ρ* = −0.244, *BF* > 100), and F (*ρ* = −0.176, *BF* > 100).

**Table 3.** Bayesian network analysis results.

Edge	Estimate (posterior)	Inclusion BF	Result
A–B	0.005	0.064	Excluded
A–C	−0.179	> 100	Included
B–C	−0.105	9.000	Inconclusive
A–D	0.000	0.031	Excluded
B–D	−0.004	0.087	Excluded
C–D	0.001	0.010	Excluded
A–E	−0.244	> 100	Included
B–E	−0.133	32.333	Included
C–E	−0.209	> 100	Included
D–E	−0.011	0.190	Inconclusive
A–F	−0.176	> 100	Included
B–F	−0.110	10.110	Included
C–F	−0.300	> 100	Included
D–F	−0.157	> 100	Included
E–F	−0.260	> 100	Included

## Discussion

The results, considered as a whole, provide a clear response: participants consistently identified the images that shared a large number of morphological criteria with the corresponding auditory stimuli. Below, we examine in more detail the potential correspondences between musical characteristics, the related morphological features (Gestalt principles), and the images more frequently chosen by participants to match with the audio stimuli.

### *Ligeti*—Atmosphères

Ligeti's *Atmosphères* (1961) is composed for a large orchestra. Its structure is static and episodic, with the orchestration designed to create an extremely dense, global sound. The texture is complex, consisting of many layered tones. The sound world of *Atmosphères* is characterised by the use of micropolyphony and clusters. Micropolyphony is a technique in which the orchestra, divided into very small sections, plays the same motif or theme in close canon, with minimal delay between entries. This creates a dense texture shaped by dynamics, register, and the musical material itself, resulting in a continuous sonic flow that evolves organically and almost imperceptibly from one section to the next.

The selected excerpt features a continuous orchestral sound that rises in pitch and then stabilises. The sound begins as a densely overlapped group of tones played by the strings at low frequencies.^6^ Gradually, the texture tightens and becomes “thinner”. Some instruments drop out (e.g., double basses, cellos, violas, and second violins), while others converge onto a few high notes (Eb7 and G7). This frequency rise is accompanied by a dynamic profile: a crescendo (from *ppp* to *ff*) leading to the highest frequency, followed by a slight diminuendo (*p*). Key aspects of this musical excerpt (Table 1) include: (i) continuity, (ii) frequency motion that is gradual, continuous, unidirectional, and closed (i.e., it stabilises at a specific point before vanishing).

**Figure 4.**
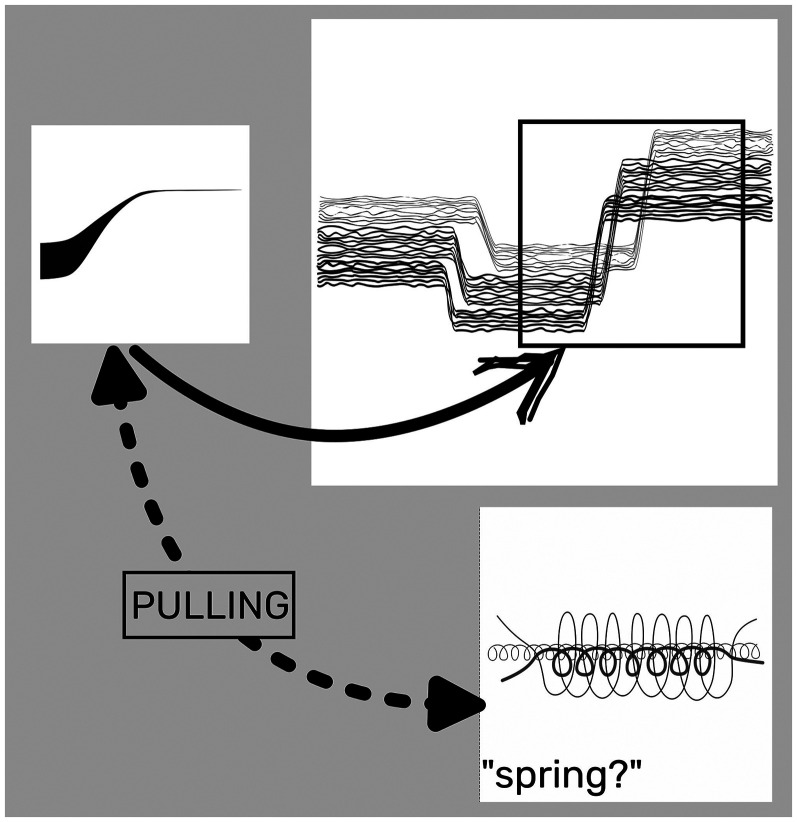
The resemblance of Image C to a spring might have influenced participants’ responses.

Besides the A image (*M* = 55.8, *SE* = 4.16), successive preferences indicate that continuity and smoothness are the most prominent criteria of selection: both images D (*M* = 42.2, *SE* = 3.36) and C (*M* = 35.4, *SE* = 2.94) (second and third choice) match with the audio excerpt in terms these criteria. If Image D may be seen as embedding Image A itself, the preference for C may stem from an unexpected response: the shape of Image C resembles a spring and evokes “an act of pulling” which may seem appropriate for representing the “thick-thin” transition from a multi-tone sound cluster at low frequencies to a high-pitched sound (Figure 4). The fourth choice, image B (*M* = 33.6, *SE* = 2.82), still satisfies the continuity criterion. However, the last two preferences, images E (*M* = 19.1, *SE* = 1.84) and F (*M* = 15.7, *SE* = 1.59)—both notably distant from the others—are characterised more by their sharp shapes than by discontinuity. Considering Image F, the least preferred, it could align with images A and D in terms of spectrotemporal arrangement (time and frequency represented along left-right and top-down axes, respectively), but it is disregarded due to its sharpness and its reversed temporal positioning, with the top line (high pitch) appearing at the beginning. Image F features a continuous stroke but with a scattered profile, and for those listeners whose attention is focused on continuity, this image may appear almost discontinuous. The pairs continuous/discontinuous and smoothness/sharpness are not entirely independent.^7^

### *Demdike Stare*—New Fakes

Demdike Stare, the Manchester-based electronic duo comprising Miles Whittaker and Sean Canty, has been active since 2009. They create tracks by layering and combining electronic samples with the raw sounds of vintage hardware, resulting in music that often evokes thick and dense atmospheres. *New Fakes* (2018) is a track from their fourth studio album, *Passion*. The selected audio extract features a continuous bass drone characterised by a distorted, grainy sound modulated through a low-pass filter. The filter gradually opens the sound to higher frequencies, following a low–high–low pattern that creates a symmetric, seemingly cyclic structure (i.e., the audio sample ends as it begins). At first glance, this pattern may evoke a process of emersion and immersion. However, the distorted and dynamic texture shifts the perception away from liquid-like qualities and may suggest a dry, grainy material instead. Key aspects of this musical excerpt include: (i) continuity, (ii) cyclic evolution (a return to the origin without transformation), and (iii) progressive change.

For this musical excerpt, participants show a slightly higher preference for image C (*M* = 50.2, *SE* = 3.84) compared to the intentionally created image B (*M* = 43.2, *SE* = 3.42). Image C, perhaps because it recalls a real-world object with a cyclic pattern (i.e., a spring), may seem to align better with the audio. Conversely, Image B, with its “hill shape,” might evoke similar real-world associations but could appear less appropriate in this context. These observations remain speculative. However, it is worth noting that the first three choices suggest that continuity and smoothness remain essential criteria for completing the task.

### *Ligeti*—Ramifications *(Version für 12 Solostreicher)*

Another of Ligeti's works, *Ramifications* (1969), exemplifies a musical aesthetic closely aligned with that of *Atmosphères*, characterised by its flexible temporality. In this piece, bar divisions in the score serve solely for synchronisation rather than as indicators of metric structure, as the work is intended to be performed with a seamless flow, free from discernible rhythm. The composition creates an impression of a heterogeneous texture, in which two groups of string instruments follow one another trying to retune each other, generating a dynamic and shifting sonic flow. The selected sample for the survey features a continuous and uniform pattern of repeated up-and-down tone movements, superimposed to form an intricate, intertwined structure. Due to the regularity of the musical material, which remains unchanged throughout the excerpt, the essential aspects that emerge are (i) regularity, (ii) continuity, (iii) stability, and a pronounced sense of (iv) recurrence (cyclicity) (Table 1). At the same time, the distinctiveness of the instrumental lines remains clearly discernible, contributing to a perception of superimposed profiles.

The designed image for this excerpt was Image C, which captures a clear set of characteristics: continuity, smoothness, regularity, cyclicity, recurrence, and layering. This image emerged as the preferred choice amongst the participants (*M* = 51.3, *SE* = 3.90). Notably, the second preference, Image E (*M* = 33.5, *SE* = 2.82), depicts repeated pseudo-rectangular shapes. Image E lacks continuity and smoothness—the primary criteria observed thus far—but shares with Image C the notions of looping and uniformity.^8^ A crucial aspect of this case is the participants’ ability to discern within the musical stimulus the singularities of various similar entities (instrumental profiles) that repeat continuously. These discrete objects are spatially organised within the internal sonic domain (i.e., figure-ground relationships) and emerge simultaneously for listeners. Images C and E convey this spatiality and instantaneity in different ways: Image C aligns with the linear (left-to-right) temporal conceptualisation and represents sonic extension as superimposed layers placed within the virtual third dimension of the image's canvas. Image E, by contrast, is chosen for its (ec)static representation: A snapshot of a moment where similar sonic profiles are visualised as equal objects placed side by side. Considering all responses, it appears that regularity (images C and E) prevails over continuity and smoothness (B, A, and D). These last three images display in fact patterns that evolve and change,^9^ whereas image F, although also transformative, lacks smoothness, explaining its low affinity with the audio sample.

### *Haas*—String Quartet n°2

Georg Friedrich Haas, a New York-based Austrian composer, primarily writes music for traditional instruments. Often associated with spectralist (or post-spectralist) composers, Haas explores microtonality and contrasts between different tuning systems (e.g., 12-TET and just intonation) within traditional orchestration, emphasising their sonic potential and impact. Haas is also known for incorporating light effects in his compositions, including works designed to be performed in complete darkness (e.g., *String Quartet n° 3 “In iij. Noct”*). His *String Quartet n° 2* (1998) belongs to an earlier phase of exploration into the sonorities created by string instruments, often featuring complex transitions of overtone chords. Excerpt #4 depicts a continuous passage where the sustained notes of the instruments are constituent of overtone chords: the cello introduces a new fundamental note and other strings follow it to rebuild every time new “sound worlds”. The transitions of the four instrumental lines, marked by slow *glissandi*, are not simultaneous, allowing overtones from different chords to coexist. This creates the sensation of interconnected parts within a unified sonic flow. During transitions, individual instruments momentarily emerge from the flow before blending back in, with the *glissando* marking these moments of transition. Key emerging criteria are (i) continuity and (ii) common fate, as instrumental lines split and merge within a unified flow. Image D was the preferred choice (*M* = 39.1, *SE* = 3.17), followed by A (*M* = 29.8, *SE* = 2.57) and F (*M* = 29.2, *SE* = 2.54), but while images D and A share a similar general pattern (with A functioning as a subset of D), image F displays a continuous sharp profile. Image F aligns with D and A for its spectrotemporal resemblance (as it could also appear as a portion of D).^10^ The musical excerpt #4, compared to others, stands out for its complexity: it evolves, changes, and alternates between a singular sonic flow and distinct instrumental lines. Consequently, it aligns with multiple images at different moments, such as F (during the first transition), A, or even B (*M* = 27.4, *SE* = 2.42), due to its smooth transitions.

### *Emptyset*—Descent

The fifth musical excerpt comes from a music track by the British electronic duo Emptyset. Their music may be ascribed to glitch electronica originated in the 90s and developed along the last two decades. By using analogue or digital devices, it is a genre of electronic music which develops short often rhythmic tracks overlaying various simple patterns and giving rise at times to intricate metric structure. On the other hand, it can show continuous or fragmented aggregates of sound typical of a detailed texture. The selected extract, from the beginning of the track, features loud, distorted electronic pulses lasting 8–10 s each. The stimulus is overwhelming in its density and saturated texture, yet its structure is simple, delineating clear sound objects with precise beginnings and endings. It is then typical of its fragmented and repetitive structure, and the distorted nature of sound may recall notions such as those of surface and its related, visual and tactile, qualities (grainy, texture, density).

In this case, numericity (the possibility of identification of a certain number of entities) is apparently predominant as a factor of choice in comparison to continuity or smoothness. The surprising absence of affinity with image F may fit in this account: the presence of a sharp profile which supposedly matches with the shapes of the spectrotemporal profile (the “sharp” attack of impulses themselves) appears not relevant as the image displays a continuous profile rather than discrete objects. Image D (*M* = 36.8, *SE* = 3.03), which shares no essential criteria with image E, is the second preference. The connection between the musical excerpt appears to lie in numericity—the identification and numbering of equal entities.^11^

### *Ligeti*—Atmosphères

The final audio extract, also taken from Ligeti’s *Atmosphères*, features a continuous high-pitched orchestral sound that abruptly transitions into a low-pitched drone. Initially, the instruments create an interlaced aggregate of sound (played *fff* by oboes and flutes). Although the sound is continuous, the abrupt change in timbre (from flutes to double basses) makes the transition feel like an interruption. As in the previous excerpt, the musical pattern is very simple and powerfully replicates the cliff-like path of the profile of image F, encompassing a double idea of frequency extent being spatially represented in verticality (up-down) and dimension (thin-thick) (“F picture is perfect. Now there is a high pitch with a drop to heavier instruments”). This clear preference seems to prevail over any general criterion that, as we observed in other cases (e.g., excerpts #1 and #3) emerges across different images.

Considering the results as a whole, some further considerations can be put forward:

1. In case of audio excerpts showing simple patterns, including single and pronounced transitions, (e.g., #5 and #6), participants select the “correct” image as their primary choice. However, all secondary preferences do not appear to follow consistent criteria and are distributed fairly evenly among the other options.
2. For more complex audio excerpts, including multiple transitions (e.g., #1, #4), participants generally identify the corresponding image as the most appropriate. Their subsequent choices suggest the presence of five criteria. The most significant is the smoothness (or sharpness) of the pattern, which, as noted earlier, overlaps with the criterion of continuity (or discontinuity). In most cases, these two criteria align. Other relevant factors include spectrotemporal resemblance, numericity (the identification of discrete entities) and symmetry/asymmetry (as regularity).
3. The interpretation of images that evoke real-world objects—such as the hill-like contour of Image B or the spring shape of Image C—may interfere with the aforementioned criteria. Participants, either consciously or unconsciously, tend to interpret these images as portraying real-world objects and thus ignoring their morphology. For instance, image B, which was designed to replicate the morphology of audio excerpt #2 (an A-B-A continuous drone filtered to higher frequencies before returning to its original texture), was selected only as a second choice. This may be due to its resemblance to a hill, a feature that has little connection to the musical episode.
4. Finally, the Western cultural bias of interpreting temporal evolution as a line from left to right and frequency vertically is an evident limitation but one that is uniformly accepted in this type of study (Küssner, 2017).

Furthermore, several intriguing questions arise concerning the specific role of vision in our task and its influence on the results obtained. For example, are the observed associative trends rooted in the unique capacity of music—particularly sound-based music—to evoke visual imagery or content? Relatedly, does this phenomenon occur because the two modalities share intrinsic similarities (e.g., Julesz & Hirsh, 1972)? Alternatively, could this be attributed to the well-documented visual dominance in humans (Hutmacher, 2019)? Another possibility is that the experimental task itself, by requiring participants to adopt an analogical mindset, encourages a visual mode of thinking and constrains their responses to visual terms. In other words, while participants might evoke olfactory or tactile mental images, they may be compelled to express their responses in visual/spatialised terms.^12^ This raises the question of whether a process of “sensory translation” occurs in the participants’ minds as they listen to and match the stimuli (Spence & Di Stefano, 2023).

Finally, some reflections on the musicological implications of our findings are warranted. Our results demonstrated consistent associations across participants, irrespective of their musical expertise or familiarity with the audio stimuli (thus excluding any potential effect of album covers on the audiovisual associations, see e.g., Venkatesan et al., 2020). This outcome may initially seem surprising given the presumed structural and formal complexity of this musical genre compared to the classical Western music repertoire. Despite sound-based music being perceptually more intricate and complex—and listeners typically having less exposure to it—participants were able to identify the expected audiovisual associations, as has been demonstrated with less complex and more traditional repertoires (Di Stefano et al., 2024, 2025). In light of Snitz et al.'s (2016) framework, we propose that while the audio stimuli exhibit complexity, they might lack intricacy, as evidenced by the highly consensual subjective ratings among participants. An additional factor that may explain the consistency of responses across different levels of musical familiarity and training is the nature of the selection itself. By drawing from a cross-genre aesthetic, the selection allows shared elements to emerge more clearly—elements that transcend stylistic differences and point to more fundamental traits, which we propose are related to morphodynamics.

## Future Directions

This article shows that sound-based music works well to study the Gestalt and morphodynamic criteria on which listeners rely in their audiovisual matchings. In future research, it would be valuable to delve deeper into the specific musical characteristics that influence the preference for one criterion over others and to investigate whether cultural diversity plays any role in shaping these preferences (e.g., limiting the reliance on the criterion of spectrotemporal dimensions). Additionally, it is important to examine more complex (e.g., longer) musical constructions to assess whether this method remains effective in cases of multiple features within complex morphodynamics (i.e., overlapping patterns evolving over time). Such investigations could provide a more comprehensive understanding of how listeners process and integrate these dynamic elements. Furthermore, the data on audiovisual associations could lay the groundwork for advancing the study of mental imagery formation, particularly in case of music-evoked visual imagery. Some of the musical and visual criteria identified in this study may serve as foundational building blocks for exploring the connection between sound patterns and the mental images they evoke.

## Conclusions

In this study, we investigated audiovisual associations within the context of contemporary and experimental music practices, particularly focusing on sound-based music. Participants were exposed to six musical excerpts and asked to rate the extent to which each audio stimulus matched with six ad hoc generated abstract images. The results suggest that smoothness (and continuity), numericity, symmetry, and spectrotemporal dimensions are the primary criteria participants rely on in their matchings for this kind of music. Findings support the idea that our fundamental crossmodal mapping of sound-based music is morphodynamic in nature. Finally, the richness of sound-based music’s structures offers opportunities to explore more diverse and nuanced criteria underlying our aesthetic engagement with sound.

## Supplemental Material

sj-docx-1-ipe-10.1177_20416695251338718 - Supplemental material for Exploring auditory morphodynamics: Audiovisual associations in sound-based music Supplemental material, sj-docx-1-ipe-10.1177_20416695251338718 for Exploring auditory morphodynamics: Audiovisual associations in sound-based music by Riccardo Wanke, Alessandro Ansani, Nicola Di Stefano and Charles Spence in i-Perception

## Notes

1. Within the overarching label of electroacoustic music, we can find compositions such as Bernard Parmegiani's *De Natura Sonorum* (1975) and Iannis Xenakis’ *Persepolis* (1971), to name just two pieces from the historical avant-garde. While the former aligns with the aesthetics of *musique concrète*, where sound objects are combined to create “pictorial” and representational musical contexts (Chion, 2009), Xenakis’ piece employs different sounds while minimising their articulation and functional roles, instead forming masses and layered constructions that emphasise intrinsic sonic features such as texture, density, and timbre. A similar distinction can be made between the works of composers such as Annette Vande Gorne, John Chowning, and Denis Smalley, on the one hand, and Alvin Lucier, Éliane Radigue, and James Tenney, on the other—all of whom can be included within electroacoustic music. At the same time, certain works across different genres, such as minimalism, spectralism or glitch electronica, share enough commonalities to form a distinct cross-genre group that we labelled here as “sound-based.”

2. To assess this, the participants first listened to a single 20 s track consisting of a mix of four stimuli representative for the genres used in our study (5 s each).

3. Conditional dependence refers to a direct relationship between two variables, determined while accounting for the influence of all other variables in the system (i.e., network). It captures unique associations that are not explained by indirect connections or shared influences.

4. This test compares the distribution of expected zeros in the data against the observed zeros.

5. The negative correlation between, for instance, images A and E on numericity criterion is based on the fact that A is a single entity (a continuous stroke from bottom to up) while E consists of three distinct entities.

6. The initial cluster spans from E3/G#3 and Db3/F3 in the sixth and seventh double basses up to harmonics of B4, C#5, D5, E5, F#5, and G5 in the other strings.

7. As in mathematics, smoothness typically refers to the differentiability of a function to a certain degree, and differentiability inherently requires continuity (Weisstein, n.d.).

8. Free comments from participants reveal this aspect: “The sound seemed similar to an insect, looping and flying chaotically over the same few notes, without ‘going’ anywhere, really (no progression)”; “Repetitive tone, like in a loop with little variation.”

9. In case of image B, the third most preferred response, the pattern returns to the origin, reinforcing the idea of cycle present in the audio sample.

10. As one of the participants observed: “The pitch increases and decreases, and I associated that with images D and A because the images also seem to increase and decrease in size. I am unsure why Image F is associated with the music, but it just felt right.”

11. One of the participants observed: “It feels like the same beat is repeated three times.”

12. Using simpler stimuli (i.e., tones/chords), Salgado-Montejo et al. (2016) created a generative task inviting participants to move a computer mouse in order to spatially represent audio stimuli.
