# Supplementary material for: Exploring auditory morphodynamics: Audiovisual associations in sound-based music
Source: Iperception. 2025 Jun 30;16(4):20416695251338718. doi: 10.1177/20416695251338718 (PMC12209565; doi:10.1177/20416695251338718)
Supplement: sj-docx-1-ipe-10.1177_20416695251338718 - Supplemental material for Exploring auditory morphodynamics: Audiovisual associations in sound-based music [file sj-docx-1-ipe-10.1177_20416695251338718.docx]

# *Exploring auditory morphodynamics: Audiovisual associations in sound-based music*

# Supplementary Materials

##


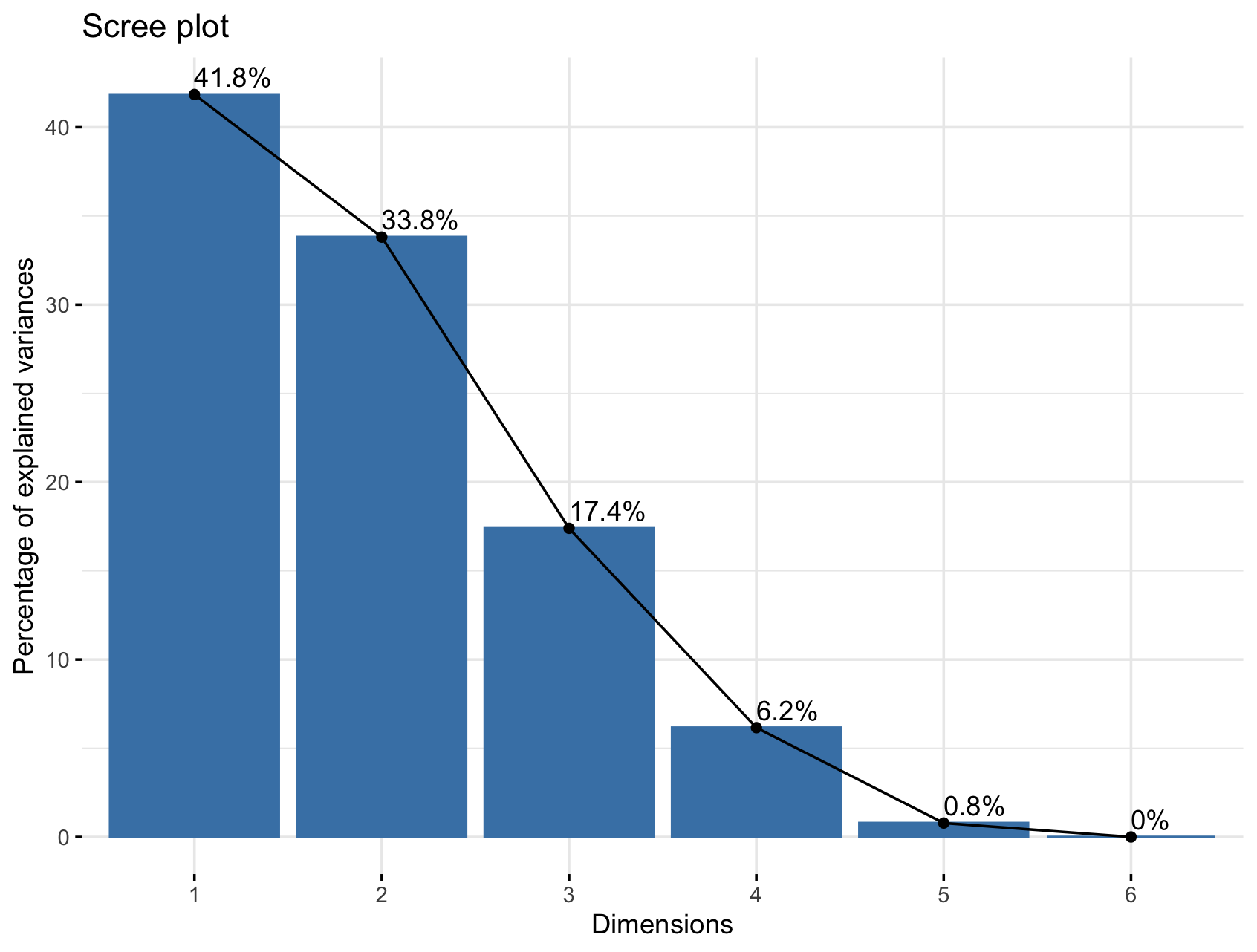


**Figure S1**. **Percentage of explained variance of the PCs (elbow plot).** A Principal Component Analysis (PCA) was conducted on the average fit score values assigned to the images for all musical excerpts. To account for the repeated-measures nature of the data and ensure an equal contribution from all participants, the fit scores were z-transformed within each participant before computing the principal components. The first two components explain 75.6% of the variance (PC_1_ = 41.8%; PC_2_ = 33.8%) [Fig. S1].


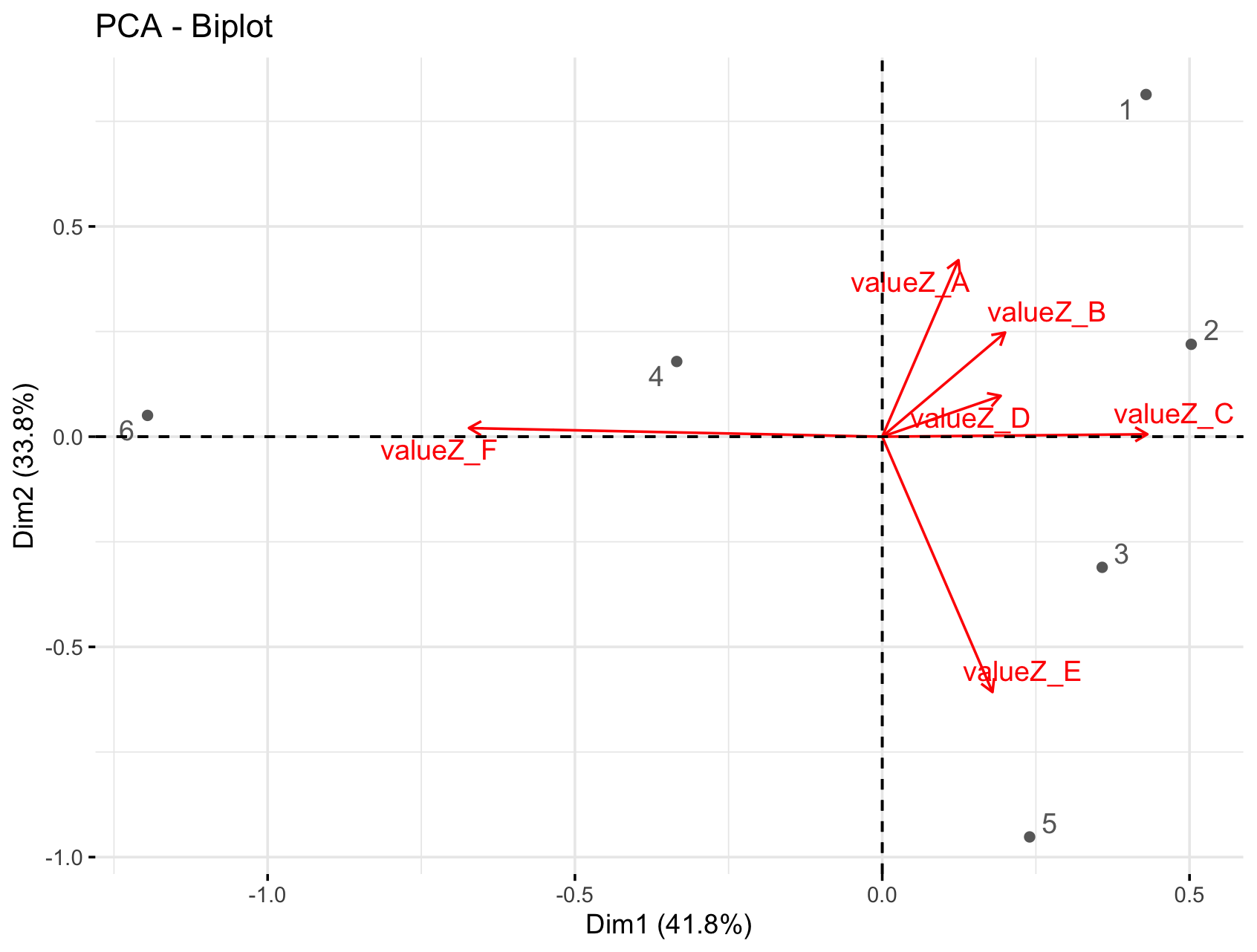
**Figure S2**. **Principal Component Analysis (PCA) Biplot.** The figure illustrates how the six musical excerpts (points labelled 1–6) are distributed within the two-dimensional space defined by the first two principal components. Each vector represents one of the six images (valueZ_A to valueZ_F) and indicates the strength and direction of its contribution to the components. Musical excerpt 1, for instance, is strongly associated with Images A (i.e., valueZ_A) and B (i.e., valueZ_B), while excerpt 6 aligns closely with Image F. This distribution reflects the differing fit score patterns across musical excerpts, thus confirming distinct image associations for each piece. Furthermore, such a representation allows us to notice the uniqueness of image F, whose scores appear to be negatively correlated with all of the others. Lastly, the score of image B is correlated with those of images D, C, and A.


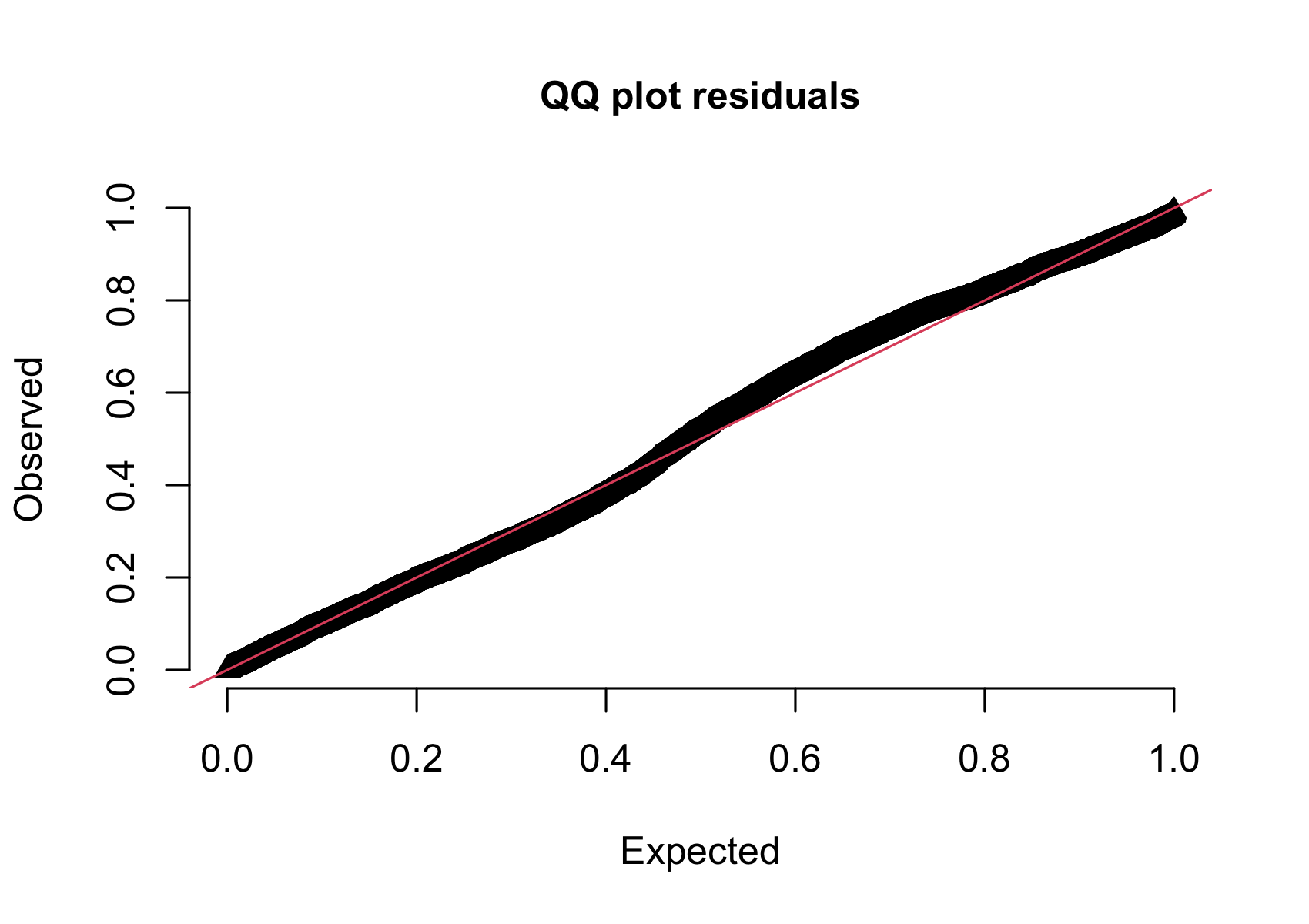


**Figure S3**. Q-Q Plot.


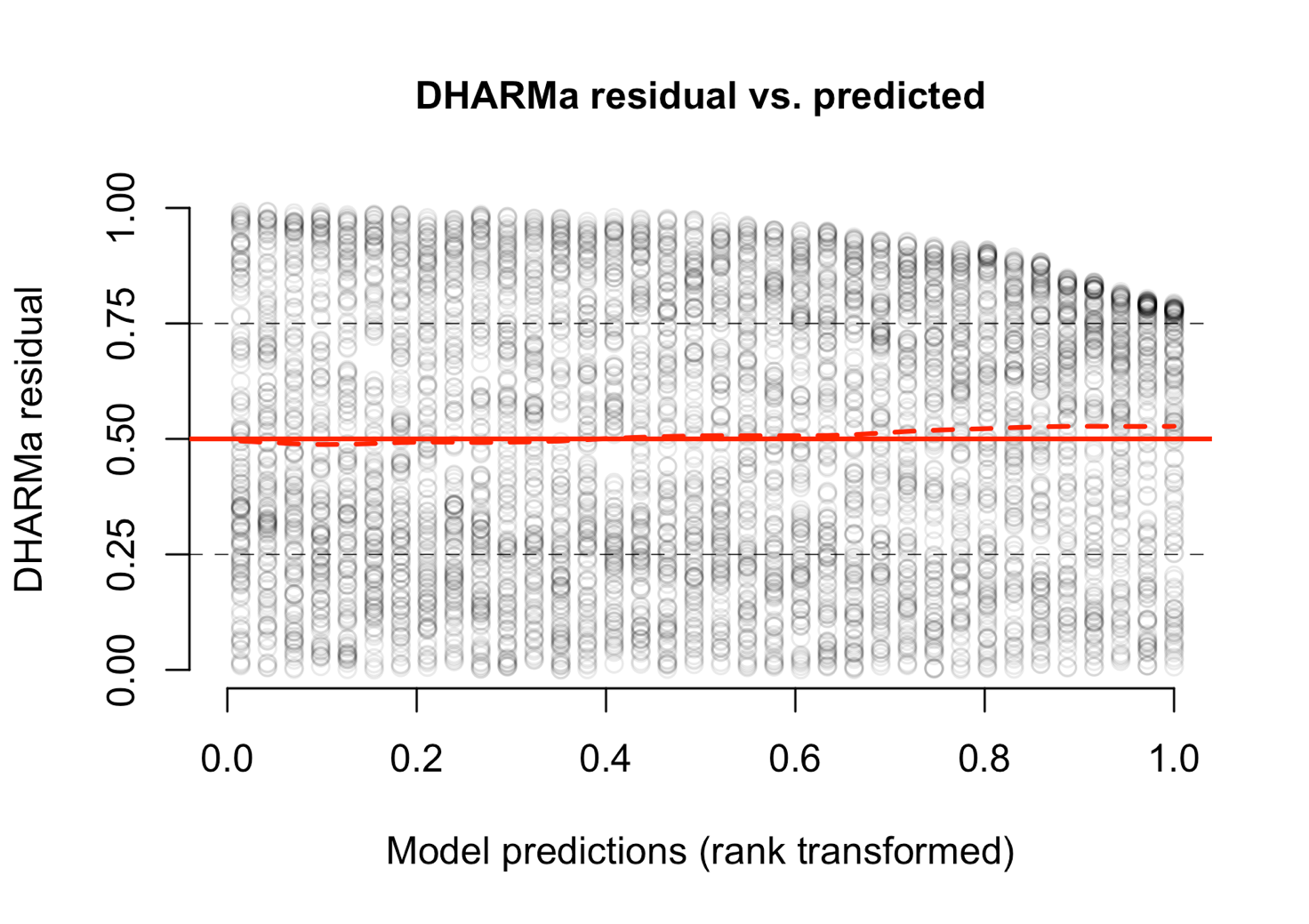


**Figure S4**. DHARMa residuals against the predicted value.


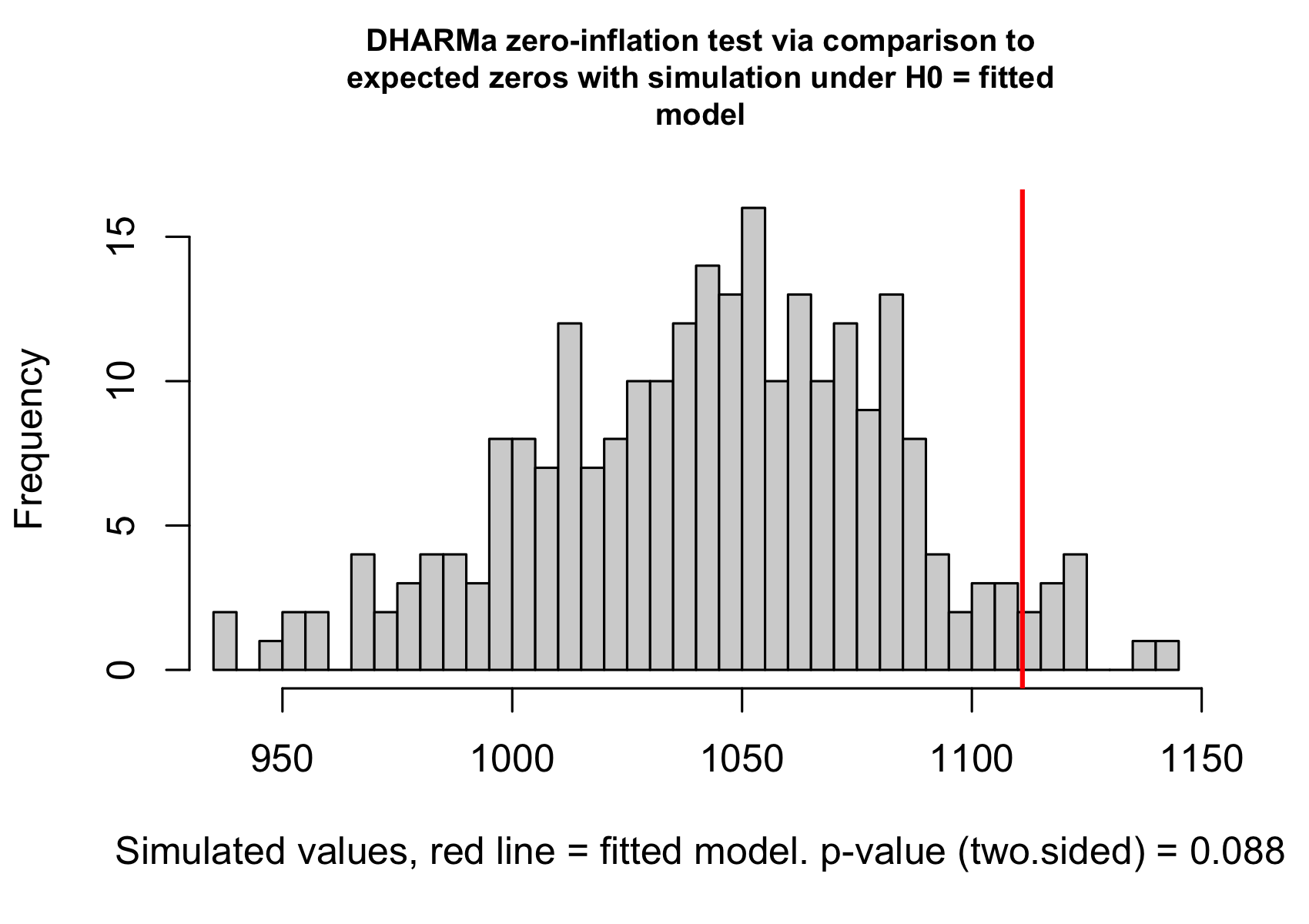


**Figure S5**. DHARMa zero-inflation test.
